# Evidence for an Auditory Fovea in the New Zealand Kiwi (*Apteryx mantelli*)

**DOI:** 10.1371/journal.pone.0023771

**Published:** 2011-08-24

**Authors:** Jeremy Corfield, M. Fabiana Kubke, Stuart Parsons, J. Martin Wild, Christine Köppl

**Affiliations:** 1 Department of Anatomy with Radiology, University of Auckland, Auckland, New Zealand; 2 School of Biological Sciences, University of Auckland, Auckland, New Zealand; 3 Institute for Biology and Environmental Sciences, and Research Center Neurosensory Science, Carl von Ossietzky University, Oldenburg, Germany; Lund University, Sweden

## Abstract

Kiwi are rare and strictly protected birds of iconic status in New Zealand. Yet, perhaps due to their unusual, nocturnal lifestyle, surprisingly little is known about their behaviour or physiology. In the present study, we exploited known correlations between morphology and physiology in the avian inner ear and brainstem to predict the frequency range of best hearing in the North Island brown kiwi. The mechanosensitive hair bundles of the sensory hair cells in the basilar papilla showed the typical change from tall bundles with few stereovilli to short bundles with many stereovilli along the apical-to-basal tonotopic axis. In contrast to most birds, however, the change was considerably less in the basal half of the epithelium. Dendritic lengths in the brainstem nucleus laminaris also showed the typical change along the tonotopic axis. However, as in the basilar papilla, the change was much less pronounced in the presumed high-frequency regions. Together, these morphological data suggest a fovea-like overrepresentation of a narrow high-frequency band in kiwi. Based on known correlations of hair-cell microanatomy and physiological responses in other birds, a specific prediction for the frequency representation along the basilar papilla of the kiwi was derived. The predicted overrepresentation of approximately 4-6 kHz matches potentially salient frequency bands of kiwi vocalisations and may thus be an adaptation to a nocturnal lifestyle in which auditory communication plays a dominant role.

## Introduction

Most birds rely heavily on their sense of hearing for communication in territorial, social and sexual contexts and for the detection of alarm signals. In a small number of species, such as barn owls (*Tyto alba*), hearing has been specialised for passive sound localisation of prey [1]. The auditory sensory epithelium of birds is the basilar papilla. Unlike the homologous mammalian organ of Corti, which is coiled and has the sensory hair cells organised in discrete rows, the avian basilar papilla is elongated but only mildly curved, with hair cells arranged in a complex mosaic (reviewed in [2], [3]). Hair cell morphology is an important determinant of frequency sensitivity in the avian basilar papilla and shows gradients both along its width and length. These morphological gradients underlie the basilar papilla's tonotopic organisation, with high frequencies being mapped basally and low frequencies apically. In particular, the morphology of the mechanosensitive hair bundles atop the cells, comprised of a group of actin-stiffened stereovilli (or stereocilia), appears to determine the basic frequency response. In all birds examined to date, systematic changes in both height and number of stereovilli along the basilar papilla have been observed [2], [4], [5], [6], [7], [8], [9], [10], [11], [12]. In those species where the tonotopic frequency representation is also known [13], [14], [15], it is mirrored quite accurately by those changes in hair-bundle morphology [12]. The most striking case is the barn owl, where hair-bundle morphology stays nearly constant in the basal half of its basilar papilla, correlated with a foveal overrepresentation of the highest frequency band between 5 and 10 kHz [7], [13]. In the present study, we exploited the known correlations between morphology and physiology in the avian basilar papilla to predict the frequency representation in the New Zealand kiwi.

In the central auditory system, the encoding of intensity and temporal information requires morphological specialisations of neuronal circuits to accurately process sound stimuli. For example, neural morphology is critical for sound localisation, a task with direct behavioural relevance for many species [16], [17], [18], [19], [20], [21], [22]. In birds, the brainstem nucleus laminaris (NL) is part of a well-characterised neural circuit for determining interaural time differences (reviewed, e.g., in [23]). The length of NL dendritic tufts varies along a rostromedial to caudolateral axis. Neurons in NL with longer dendrites found in more caudolateral regions are tuned to lower best frequencies and those with short dendrites are found in more rostromedial regions and tuned to higher best frequencies [22], [24], [25], [26], [27], [28]. In chicken and emu, where NL dendritic lengths have been carefully examined, a smooth and linear increase in dendritic length from rostromedial to caudolateral is seen [22], [24], [25]. In the chicken, this mirrors the known physiological tonotopic gradient [29]. In the emu, a similar correlation holds, on the assumption that the tonotopic representation of the basilar papilla persists in the NL [22]. Again, the barn owl provides an interesting exception. Instead of being bitufted, NL cells in the barn owl have numerous short dendrites distributed around the soma [28], [30], [31]. This derived organisation of NL is thought to have arisen in association with the barn owl's particularly acute high frequency hearing (up to 10 kHz), its sensitivity across a wide range of frequencies and its enhanced ability to encode auditory temporal cues for precise sound localisation [1], [32], [33], [34], [35].

The auditory specialisation in the barn owl has likely evolved in parallel with a shift to a nocturnal niche. Bird species that occupy a nocturnal niche are extremely rare and some, such as the barn owl, may have evolved specialisations to function at low light levels. Thus, together with an auditory specialisation, barn owls also have a well developed visual system, consisting of large eyes, a specialised retina and large brain areas for processing visual information [36], [37], [38], [39], [40], [41], [42], [43]. The New Zealand kiwi (*Apteryx spp*.) has also made a shift to a nocturnal niche, but also to a ground-dwelling one. However, kiwi probably do not rely much on vision to function in their nocturnal environment [44] and therefore may be expected to rely on other sensory modalities, namely olfactory, tactile and/or auditory. Indeed, there is some evidence, both anatomical and behavioural, that the kiwi olfactory and tactile systems are particularly well developed [44], [45], [46], [47], [48], [49], [50], but the possibility of an auditory specialisation has not been considered.

We have examined both the inner ear (basilar papilla) and auditory brainstem (NL) in the kiwi and have found that their auditory system shows specialisations associated with an overrepresentation of high frequency coding that originates in the cochlea and is preserved in the auditory brainstem.

## Results

**Figure 1:**
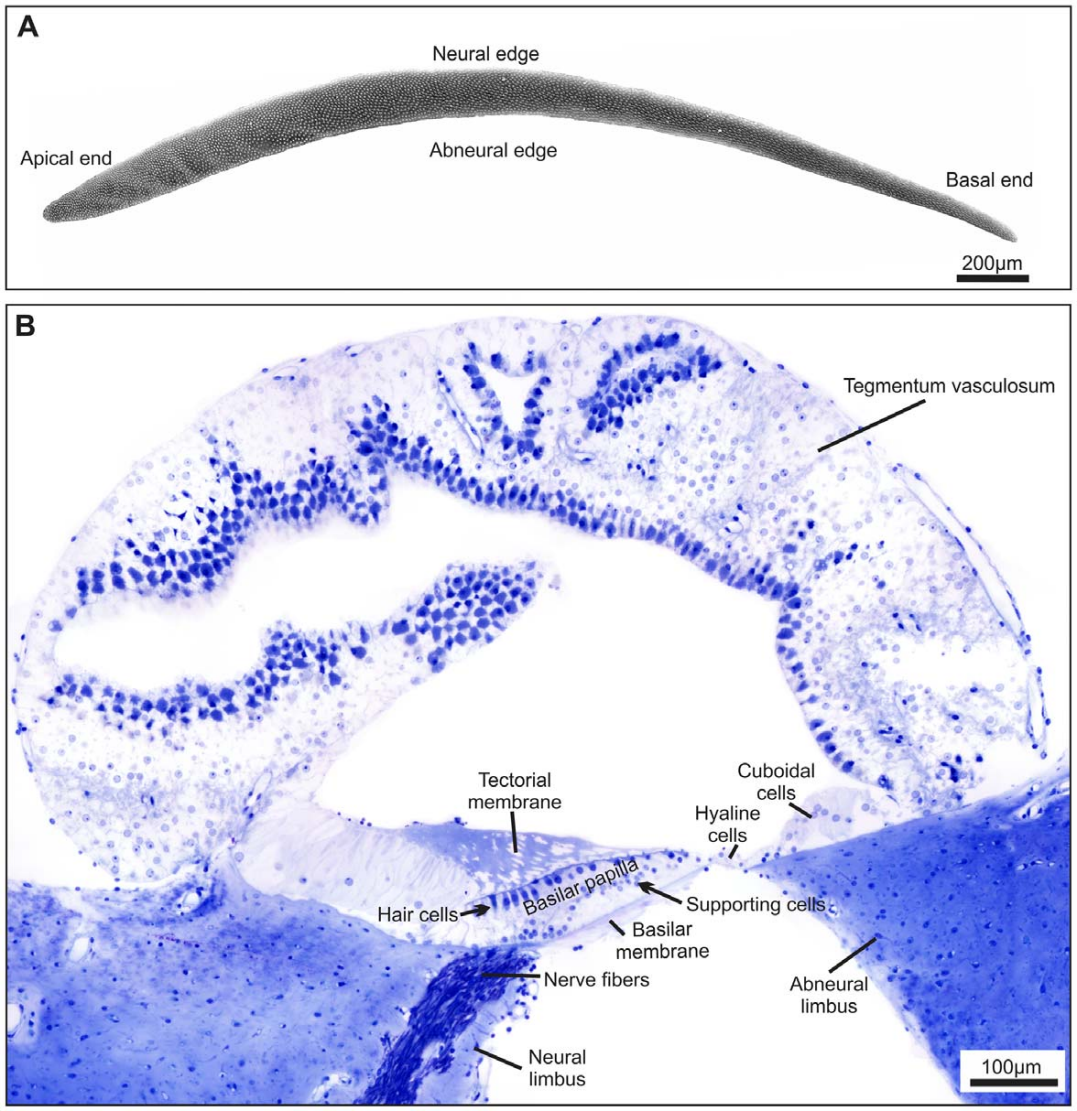
Overview of the kiwi basilar papilla. A: Surface view of the basilar papilla obtained from scanning electron microscopy. The tiny white dots represent individual hair-cell bundles. B: Cross section of the kiwi cochlea approximately half way along the basilar papilla. Key structures are labelled.

The kiwi basilar papilla was approximately 4 mm long (corrected for tissue shrinkage), which is representative for a bird of its size [3]. It showed the typically avian, moderate curvature and tapered width (Fig. 1). However, the papilla was unusually slender, being only about 200 µm across at its widest point in the apex, and narrowing to about 70 µm near the basal end (corrected for tissue shrinkage). Correspondingly, the total hair-cell count was moderate, at about 4000 (n = 1).

**Figure 2:**
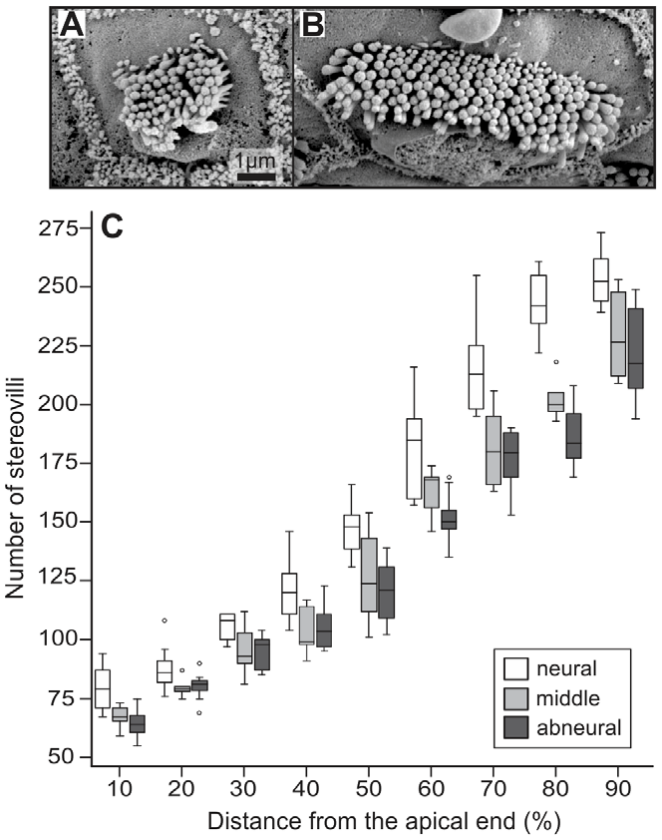
Number of stereovilli in mechanosensory hair-cell bundles increased nearly linearly along the basilar papilla. A, B: Two examples of SEM micrographs of hair bundles located at 10% from the apical end, neurally (A) and at 90%, abneurally (B). C: Boxplot of stereovillar numbers as a function of papillar position. For each longitudinal position, 3 different values are shown for hair cells located at the neural edge, near the midline and at the abneural edge, respectively.

In kiwi, the number of stereovilli per hair-cell bundle changed systematically along the length of the basilar papilla (Fig. 2). A total of 237 hair cells from 3 basilar papillae were evaluated. Median values were 69 stereovilli for hair bundles located near the apical end and 241 for hair bundles near the basal end of the basilar papilla. Hair cells in neural positions contained significantly more stereovilli than both medial and abneural hair cells (Kruskal-Wallis and subsequent Mann-Whitney U-tests, all p<0.01; Fig. 2).

**Figure 3:**
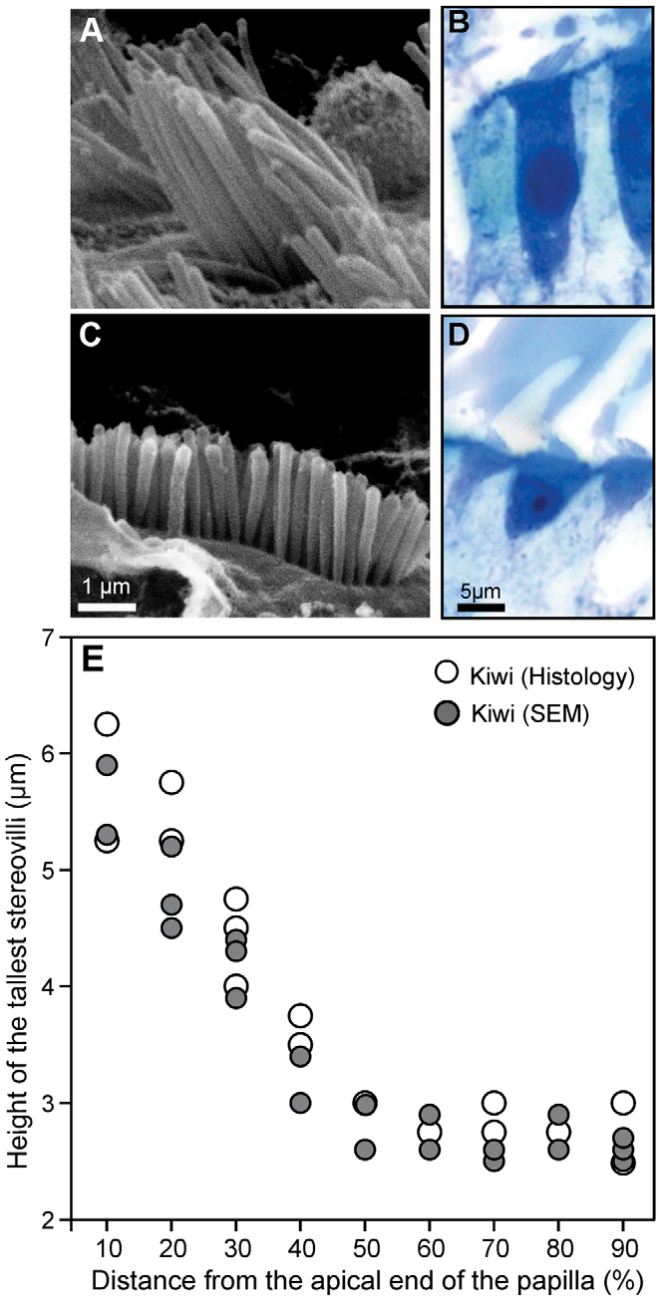
Height of the mechanosensory hair-cell bundles was nearly constant along the basal half of the basilar papilla. A, C: SEM micrographs of individual hair bundles from an apical location (A; 10% from the apical end) and a basal location (C; 80% from apex). Note that in C, the view is directly perpendicular to the tallest row of stereovilli, which obscures the shorter rows. B, D: Two examples of histological sections of individual hair cells from an apical location (B; 20% from the apical end) and a basal location (D; 90% from apex). E: Height of the tallest stereovilli in individual, neurally located hair cells, as a function of longitudinal papillar position. Different symbols show measurements from an SEM specimen (corrected for 25% shrinkage) and a series of light-microscopical sections, respectively.

The gradient in stereovillar height within a bundle was typical of birds, with the tallest stereovilli generally facing toward the abneural edge (Fig. 3A–D). The height of the tallest stereovilli in kiwi hair cells ranged from ∼6 µm at the apical end to ∼3 µm at the basal end of the papilla (Fig. 3). Measurements taken from one SEM specimen (n = 28) were consistent with those from light-microscopical sections (n = 26). Stereovillar height gradually decreased from the apical end to approximately half way along the papilla, after which it remained nearly constant (Fig. 3E).

**Figure 4:**
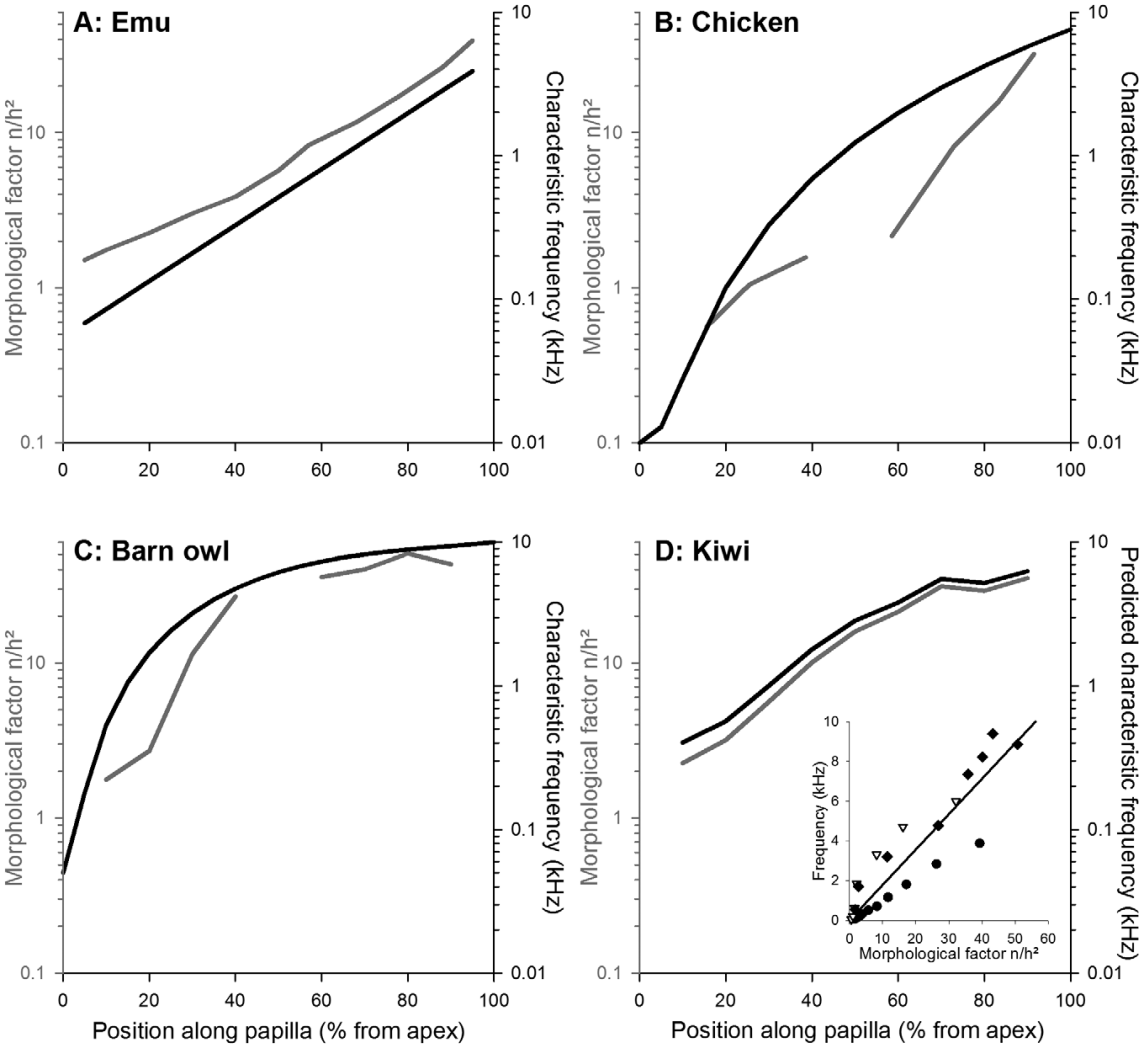
Prediction of basilar-papilla frequency map derived from hair-bundle morphology. A–C: Known frequency representations along the basilar papilla of the emu (A), chicken (B) and barn owl (C) compared to the variation in morphological factor (see Methods). Frequency maps were plotted using the equations of [14], [63] and an improved polynomial fit to the data of [13]; they are shown in black, referring to the right ordinates. Stereovillar height and number for neurally-located hair cells were taken from [7], [11], [12], [64], [65] and the morphological factor derived from those is shown in gray, referring to the left ordinates. Note that the morphological factor correlates well with the species-specific shape of the frequency maps. D: Morphological factor and a prediction for the frequency distribution in the kiwi. The prediction is based on a linear regression of frequency as a function of morphological factor for the pooled data from emu (circles), chicken (triangles) and barn owl (diamonds), shown in the inset.

Based on the height of the tallest stereovilli and the number of stereovilli/bundle for neurally located hair cells, we derived an estimated frequency map for the kiwi basilar papilla (Fig. 4A; see Methods for details). The most striking feature of this prediction was a pronounced overrepresentation of a narrow frequency band over the basal third of the papilla. A more conventional logarithmic increase in frequency was predicted along the apical two-thirds of the basilar papilla. In terms of the specific best frequencies represented, we estimated an upper limit of approximately 6 kHz and a lower frequency limit at about 0.4 kHz. The spatially overrepresented band was approximately 4 to 6 kHz.

**Figure 5:**
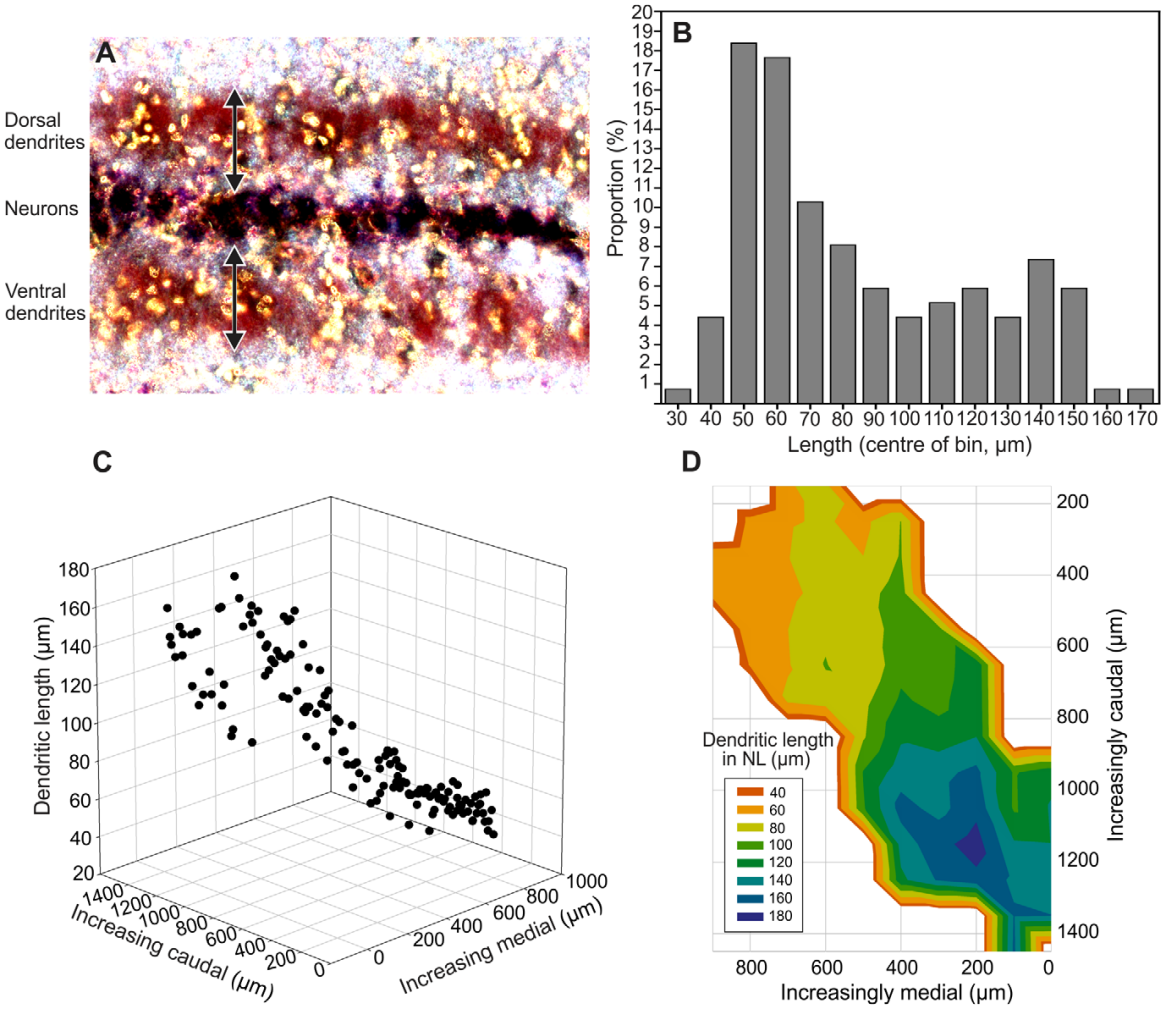
Dendritic length of neurones was nearly constant in the rostromedial half of nucleus laminaris. A: Image of a section of nucleus laminaris (NL) showing the dorsal and ventral dendritic neuropil as indicated by the arrows. B: Percentage of each dendritic length in NL, classified into 10 µm bins: C, D: Three-dimensional plots of dendritic length as a function of both rostrocaudal and mediolateral position within NL. In D, median dendritic lengths for all cells located in a 10×10 µm square (n = 1 to 5) are plotted as a flat topographical map and colours represent dendritic lengths as shown in the legend. Note that the orange fringe (suggesting short dendrites) around caudal positions is an artefact of the plotting procedure. Note the regular decrease of dendritic length in the caudal half of the nucleus and short dendrites of nearly constant length throughout the rostromedial half.

The distribution of dendritic lengths throughout NL was obtained from measurements of both dorsal and ventral dendritic neuropils at regular intervals along NL (Fig. 5A). As dorsal and ventral neuropils did not differ significantly in length (Wilcoxon paired-sample test, p = 0.47, n = 136), the average value was used for each evaluated cell. Dendritic length ranged from ∼30–180 µm (Fig. 5B). The largest proportion of cells had dendritic lengths between 50–60 µm with nearly half of all dendrites being between 50-70 µm long (Fig. 5B). There appeared to be two peaks in the number of cells with given dendritic lengths, with the major peak at shorter dendritic lengths (∼50–60 µm) and the other, much smaller peak, at longer values. Topographically, the most interesting feature of the dendritic length in NL was that it remained relatively constant within the rostromedial half of NL (Fig. 5C, D). Here, dendritic length showed little or no changes along either the mediolateral or rostrocaudal axes.

## Discussion

In both the inner ear and brain of the kiwi there was a conspicuous overrepresentation of features known to be correlated with the processing of higher frequencies. In the kiwi basilar papilla, stereovillar height did not map in a simple linear manner against longitudinal position as it does in most birds (reviewed in [3]). Instead, stereovillar height remained largely unchanged in the basal half. This pattern is similar to that seen in the barn owl, but is not typical of any other bird, including kiwis' closest relatives, the emu and rhea [8], [12]. Functionally, this unusual feature led to the prediction of an overrepresentation of a narrow band of frequencies – an auditory fovea - comprising the upper end of the kiwi's range. The underlying assumption that frequency tuning is reasonably predicted by micromechanical parameters was verified by comparisons with independently derived frequency maps for three other bird species (Fig. 4). While micromechanical tuning is known to be supplemented by electrical tuning (variations in the hair cells' ion-channel properties) in birds, both mechanisms are likely to be closely matched in frequency and the known papillar frequency maps on which our estimate for the kiwi is based would naturally be the combined result of both tuning mechanisms. Furthermore, electrical tuning is probably of only marginal significance in the upper hearing range of birds (reviewed in [3], [51]). Our prediction for the kiwi's frequency representation, especially the unusual higher-frequency fovea, should thus be robust against distortion due to contributions by electrical frequency tuning.

Similar to the basilar papilla, NL in the auditory brainstem suggested an overrepresentation of the upper hearing range in the kiwi. In contrast to what is found in chicken and emu [22], [24], [25], the dendritic gradient in the kiwi NL did not show a simple linear pattern in the rostromedial-caudolateral direction. Instead, dendritic length decreased along the putative frequency axis in more caudolateral regions, but remained relatively constant towards the rostromedial, high-frequency end of the nucleus.

Among birds, such a foveal frequency representation has to date only been found in barn owls. Very similar to the kiwi, the barn owl shows a lack of morphological gradients within the basal half of the basilar papilla and numerous short dendrites in the rostromedial regions of its NL [7], [31], [52]. In the owl, these features correlate with a massive overrepresentation of responses to high frequencies between 5 and 10 kHz in the same regions [7], [13] which is thought to reflect the behavioural importance of that frequency band for precise sound localisation of prey [32].

**Figure 6:**
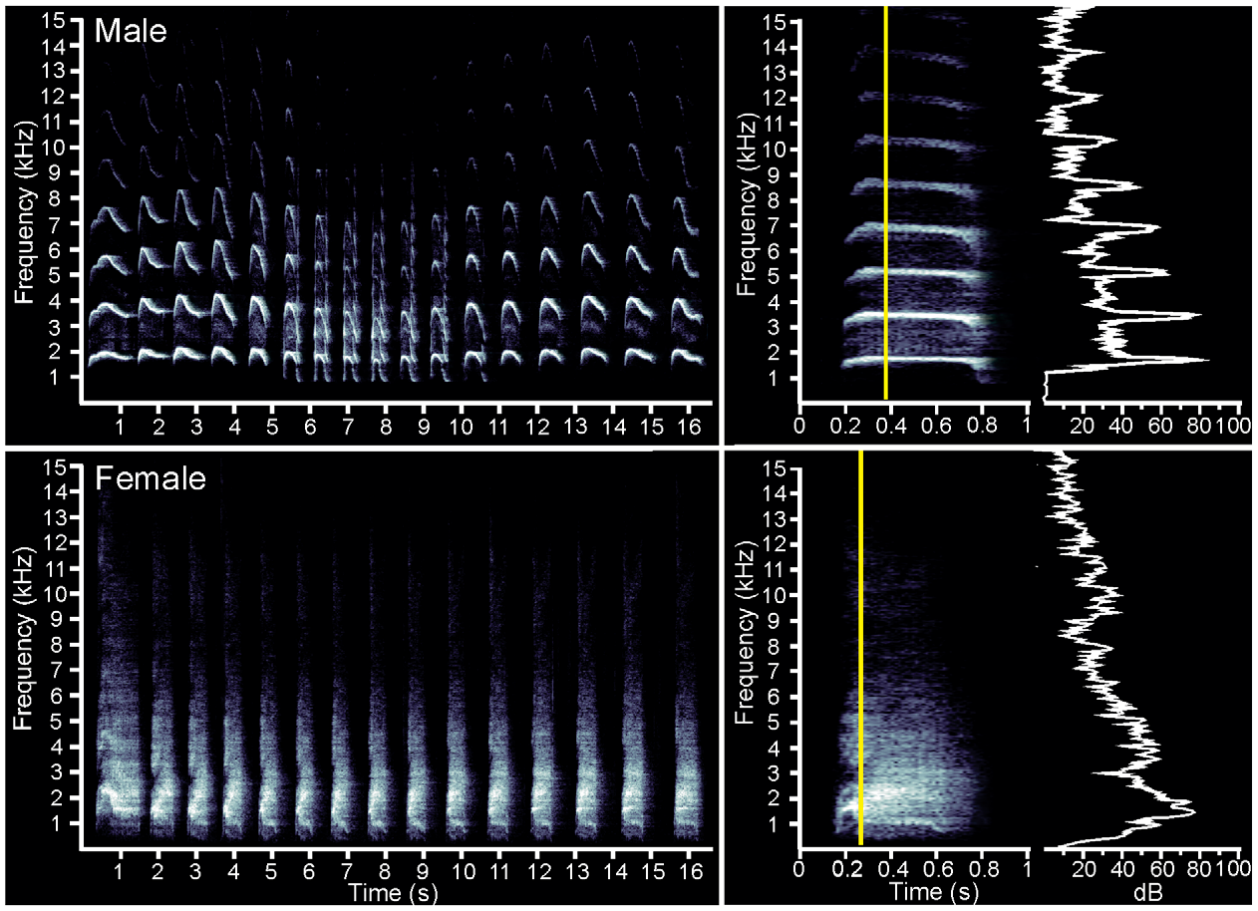
Spectrograms of typical male and female kiwi vocalisations. The entire calls are shown to the left and an enlargement of one component of the calls with a power slice of the area indicated by the yellow line to the right. For details regarding recording and analysis methods see [54]. Briefly, spectrograms and power spectra were produced with a Fast Fourier Transformation (FFT) size of 1,024 points using a Hamming window and 50% overlap, which produced a frequency resolution of 56 Hz.

The predicted frequency representation in the kiwi is clearly unusual and is thus likely to be an adaptation to the nocturnal and ground dwelling ecological niche that kiwi have come to occupy. Kiwi are known to call at night and their most common vocalisation, the whistle call, has been physically characterised [53], [54]. While male and female calls differ significantly, both contain prominent high-frequency components in the 2-6 kHz range (Fig. 6). It has not been rigorously tested whether kiwi are able to identify individual vocalisations; however, apparent duetting behaviour has been observed between established pairs [54], [55] suggesting that they can. The rich harmonic structure and frequency modulations of the call of the male and formant structure in the call of the female, in particular, provide potential cues for individual recognition within the frequency band that we predict to be overrepresented in kiwi hearing.

While conspecific vocalisations may be the most obvious match to the kiwi's suggested hearing specialisation, other auditory functions should not be disregarded. The New Zealand native forest houses earthworms and many large nocturnal insects, including beetle larvae (Coleoptera) and cicada nymphs (Hemiptera) that kiwi are known to feed on [56], [57], [58] and that are likely to produce rustling sounds in the leaf litter akin to those of mice that are hunted by owls [59]. Some of these nocturnal insects also produce communication calls [60] that could be used by kiwi to locate them. Thus, kiwi may be using hearing to detect prey in a similar way to the barn owl.

It is clear from findings in this study that the auditory system of kiwi has evolved in a manner quite unlike that of most other birds and shares many similarities with that of the barn owl, another avian auditory specialist. The driving force behind their auditory specialisations is likely to be the nocturnal lifestyle that both species have adopted and which is rare among birds. Indeed, functioning in this niche requires very different sensory strategies compared to those used by diurnal birds. Thus, the findings in this study of the auditory system, although unexpected, accurately reflect the kind of sensory challenges that are faced by kiwi when functioning in their natural environment.

## Materials and Methods

The data reported here are derived from 4 North Island brown kiwi specimens (*Apteryx mantelli*; one adult and 3 juveniles, the juveniles ranging in age from 1 week to 3 months). Experiments were carried out under research permits from the New Zealand Department of Conservation # NO-16732-FAU, NO-16732-RES, NO-18095-DOA. All specimens were provided to us dead by conservation authorities and wildlife veterinarians and thus no further ethics approvals were required to undertake this research. Three of the birds were provided freshly dead and were immediately fixed by transcardial perfusion using 4% paraformaldehyde (PFA) in 0.1 M phosphate buffer. One juvenile individual was euthanased by a veterinarian, its head removed, transported under cooling and immersion-fixed in 4% PFA within 2 hours of death. All tissue was stored in 4% PFA until processed further.

Kiwi are precocial birds and hatch fully developed and equipped to survive independently in their environment. We therefore assume that the morphology of the ear in juvenile kiwi is in most respects adult-like and the data reported here reflect the mature condition. This is supported by studies in the emu, a close relative of the kiwi, which found that the morphology of the basilar papilla in hatchlings showed only small differences to that in adults [12]. As for the dendrite lengths in NL, all previous studies were carried out in hatchlings (e.g., [22], [24]) and it is unknown whether dendritic lengths are adult-like at this age. However, for this study we were only interested in the length gradients and we do not predict best frequency based on dendritic length. Therefore, we are assuming that the age of the kiwi should not influence the length gradients.

### Light microscopy of the basilar papilla

Cochleae were dissected free of bone and postfixed in 1% OsO_4_ in PBS. One kiwi cochlea obtained from the 1-week old chick of unknown sex was then dehydrated in a graded series of ethanol concentrations and embedded in epoxy resin (“Durcupan” by Sigma-Aldrich, NSW 2154, Australia) via ascending concentrations in propylene oxide. The resin was prepared as a soft variant for semi-thin sectioning (components A/M : B : C : D at 24.5 : 17.8 : 1 : 3.8 parts by weight). The specimen was completely and serially cut into 5 µm-thick cross sections and stained with toluidine blue. The angle of sectioning was adjusted in areas of pronounced papilla curvature to maintain a section plane orthogonal to the long axis of the basilar papilla throughout. At 10%-intervals along the basilar papilla, three neighbouring sections were selected for detailed analysis. The sections were digitised using a Nikon Digital Sight cooled colour camera attached to a Leica DMR upright microscope and an oil immersion 100x objective (objective N.A. =  1.4; condenser N.A.  =  0.9). The height of the tallest stereovilli was measured from the neural-most hair cell from each image using ImageJ (http://rsbweb.nih.gov/ij/).

### Scanning electron microscopy (SEM) of the basilar papilla

Three kiwi cochleae (one adult male, one juvenile female and one juvenile male; juveniles were between 2–3 months old) were used for SEM. Cochleae were dissected free of bone and lightly stained with Janus Green to visualise the tectorial membrane. The tegmentum vasculosum, tectorial membrane, lagenar otolith and overhanging parts of the lagena were then carefully dissected away with fine forceps. Specimens were postfixed in 1% OsO_4_ in phosphate-buffered saline (PBS), dehydrated, critical-point dried in carbon dioxide (Baltec CPD 030), mounted on stubs and sputter-coated with a thin layer of gold. Specimens were viewed using a Philips XL30S FEG (University of Auckland, New Zealand) or a Zeiss ULTRA plus (University of Sydney, Australia) scanning electron microscope at 5 and 20 kV acceleration voltage, respectively. The entire basilar papilla was first documented as a series of photographs while tilting the SEM stage to maintain a perpendicular view of the papilla's surface. These provided a virtually flattened photomontage of the entire structure (final print magnification of 800×) that was used to determine standard positions at regular 10%-intervals along the papilla. Hair-bundle morphology was then evaluated at these positions at higher magnification. For stereovillar counts, the specimen was tilted for a view perpendicular to the papilla's surface and 9 hair cells were evaluated at each papillar position, three each from the very neural edge, the approximate midline and the very abneural edge. For measurements of stereovillar height, the tilt was adjusted orthogonal to the bundle's tall axis. Due to the limitations of SEM stage movement, stereovillar heights could only be obtained for a restricted sample of hair cells in one specimen, all of which were situated near the neural edge of the basilar papilla. Calibration specimens with known grid spacing were used to verify correct calibration of SEM images. SEM measurements were corrected for 25% shrinkage [61].

### Derivation of an estimate for the basilar papilla's tonotopic map

Stereovillar height (h) and number (n) are two major determinants of a hair cell bundle's micromechanical frequency response. A morphological factor, n/h^2^, was therefore defined which reflects the input of those two parameters into calculations of passive resonance frequency (e.g., [62]). The variation in this morphological factor along the basilar papilla predicted well the shape of the independently known frequency representations in the emu, chicken and barn owl (Fig. 4A–C). When plotting known characteristic frequency for those 3 species as a function of the morphological factor, the relationship was well fitted by a linear regression through the origin (inset in Fig. 4D):

frequency (kHz)  =  0.1781 * morphological factor

This equation was then used to predict a frequency representation along the basilar papilla of the kiwi on the basis of stereovillar height and number.

### Measurements of dendritic lengths in brainstem sections

Dendritic measurements from kiwi NL were obtained from a juvenile kiwi approximately 2–3 months of age. The brain was cryoprotected in 30% sucrose in 0.01 M PBS and placed in a solution of 15% gelatine with 30% sucrose at 40°C for one hour. The brain was placed in a custom-made mould so that independent fiduciary points could be made in the gelatine for later alignment of tissue sections (see below). A PBS solution containing 15% gelatine, 30% sucrose solution and black fabric dye (to darken the gelatine solution) was then poured over the brain. Once set, the gelatine block, including the brain, was removed, trimmed and placed into 4% PFA overnight. The block was sectioned on a sliding freezing microtome at 50 µm thickness in the sagittal plane. Sections of NL were stained with cresyl violet and imaged using a Nikon Eclipse 80i light microscope and camera and a 20x objective (N.A.  =  0.5). The dendritic neuropil of NL could be visualised using dark field filters. For each image the entire rostrocaudal extent of NL was measured along the cellular chain, from the most caudal cell to the most rostral cell in the image. For each cell, its distance from the most caudal cell was determined, and the linear extent of its ventral and dorsal dendritic neuropil was measured. Measurements were only obtained from cells that were clearly positioned within the monolayer part of NL. To reconstruct all cell positions along a common rostrocaudal axis, the position of NL's rostral pole relative to the fiducial points was determined for each section. The most rostral point over all sections was then defined as rostrocaudal  =  zero and all other rostrocaudal positions recalculated accordingly.
